# Adjacent cationic–aromatic sequences yield strong electrostatic adhesion of hydrogels in seawater

**DOI:** 10.1038/s41467-019-13171-9

**Published:** 2019-11-12

**Authors:** Hailong Fan, Jiahui Wang, Zhen Tao, Junchao Huang, Ping Rao, Takayuki Kurokawa, Jian Ping Gong

**Affiliations:** 1 Institute for Chemical Reaction Design and Discovery (WPI-ICReDD), Hokkaido University, N21W10, Kita-ku, Sapporo 001‐0021 Japan; 2 Faculty of Advanced Life Science, Hokkaido University, N21W11, Kita-ku, Sapporo 001-0021 Japan; 3 Graduate School of Life Science, Hokkaido University, N21W11, Kita-ku, Sapporo 001-0021 Japan; 4 Global Station for Soft Matter GI-CoRE, Hokkaido University, N21W11, Kita-ku, Sapporo 001-0021 Japan

**Keywords:** Polymer chemistry, Gels and hydrogels

## Abstract

Electrostatic interaction is strong but usually diminishes in high ionic-strength environments. Biosystems can use this interaction through adjacent cationic–aromatic amino acids sequence of proteins even in a saline medium. Application of such specific sequence to the development of cationic polymer materials adhesive to negatively charged surfaces in saline environments is challenging due to the difficulty in controlling the copolymer sequences. Here, we discover that copolymers with adjacent cation–aromatic sequences can be synthesized through cation–π complex-aided free-radical polymerization. Sequence controlled hydrogels from diverse cation/aromatic monomers exhibit fast, strong but reversible adhesion to negatively charged surfaces in seawater. Aromatics on copolymers are found to enhance the electrostatic interactions of their adjacent cationic residues to the counter surfaces, even in a high ionic-strength medium that screens the electrostatic interaction for common polyelectrolytes. This work opens a pathway to develop adhesives using saline water.

## Abstract (web summary)

Specific sequences are essential for the development of cationic polymers that can adhere to negatively charged surfaces in saline environments. Here, the authors show that copolymers with adjacent cation–aromatic sequences can be synthesized through cation–π complex-aided free-radical polymerization, which exhibit fast, strong, but reversible adhesion.

## Introduction

Developing adhesives functioning in marine environment is a great challenge. Mimicking sessile marine organisms is an efficient way. For example, catechol-based adhesives inspired from mussel have been extensively developed in the last decade^1,2^. However, the performance of such adhesives in marine environments is still barely satisfactory, because the catechol group is easily oxidized to lose adhesion^3–5^. In aqueous environment, many solid surfaces, including rocks, glasses, and metals, are negatively charged^6^. Thus, as an alternative strategy, electrostatic interaction could be utilized as a major mechanism for adhesives to these surfaces^2,7^. However, in saline water, especially at high ionic-strength conditions like seawater, the electrostatic interaction between oppositely charged surfaces normally diminishes due to the Debye screening effect^6^. Biological systems provide us a molecular clue to solve this problem^8–11^. Adjacently located amino acids of cationic and aromatic residuals have been found to enable proteins to adhere on negatively charged bilayer membranes through electrostatic interaction^12,13^. Thus, the cationic–aromatics sequence provides a molecular design model to develop adhesive hydrogels or glues for negative surfaces in saline solution. However, there is a wide knowledge gap between the molecular design, e.g. adjacent cationic–aromatic sequence, and development of materials as sequence-controlled radical polymerization is still a central challenge, and there are few efficient, scalable, and cheap methods for bulk material formation^14,15^. For example, free-radical polymerization is a highly scalable and cost-effective method, but this traditional one-pot method usually does not permit precise monomers sequence control, except for a few specific monomer combinations^16,17^. In contrast, the state-of-the-art polymerization methods for precise control of monomer sequences are generally multi-step, have a low product yield, and are expensive^18–20^.

In this study, we discover that diverse pairs of cationic and aromatic monomers form copolymers with adjacently located cationic and aromatic sequence, hereinafter referred to as poly(cation-*adj*-π) (adj is short for adjacent and π for aromatic monomer), by the traditional one-pot free-radical polymerization. The poly(cation-*adj*-π)s are water soluble and can form strong and self-recoverable physical hydrogels in seawater. These hydrogels also exhibit strong but reversible adhesion to negatively charged surfaces in seawater based on electrostatic interactions. The sequence-controlled poly(cation-*adj*-π)s that can be synthesized in abundance should have promising potential applications, such as glues for undersea leakage, sea sand binders for preserving marine environment, coagulants for concrete-making under-seawater, etc.

## Results

### Adjacent sequence formation of poly(cation-*adj*-π)

**Fig. 1:**
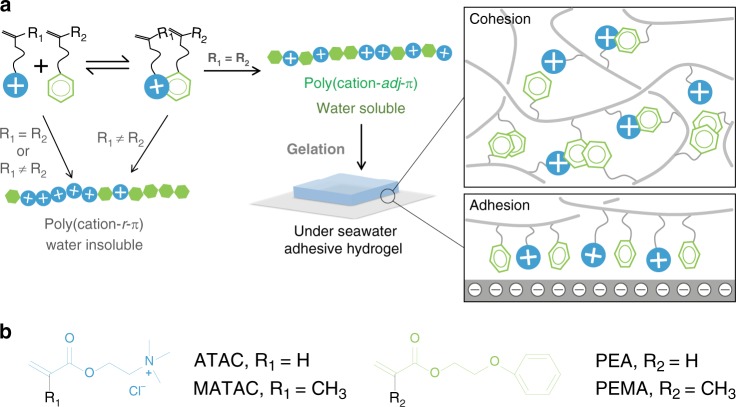
Schematic illustration of design strategy. **a** Cation–π complex-aided free-radical polymerization to synthesize poly(cation-*adj*-π) with adjacent cationic–aromatic sequences and its supramolecular hydrogel showing electrostatic adhesion in seawater (adj is short for adjacent and π for aromatic monomer). The counter ions and salt ions are not included for simplicity. **b** Chemical structures of four typical monomers are used in this study. Cationic monomer: 2-(acryloyloxy)ethyl trimethylammonium chloride (ATAC), 2-(methacryloyloxy)ethyl trimethylammonium chloride (MATAC); and aromatic monomers: 2-phenoxyethyl acrylate (PEA), 2-phenoxyethyl methacrylate (PEMA)

There are two prerequisites for the formation of such poly(cation-*adj*-π)s with controlled sequences: formation of cationic/aromatic complex by cation–π interaction in the precursor solution of polymerization, and the same reactive vinyl head (R_1_ = R_2_) of cationic and aromatic monomer pairs (Fig. 1a).

As typical examples, we show the results using two cationic monomers of quaternary-N type (2-(acryloyloxy)ethyl trimethylammonium chloride (ATAC) and 2-(methacryloyloxy)ethyl trimethylammonium chloride (MATAC)) and two aromatic monomers of phenyl-end (2-phenoxyethyl acrylate (PEA) and 2-phenoxyethyl methacrylate (PEMA)) that have different derivatives in their reactive vinyl heads (R_1_, R_2_ for cationic and aromatics, respectively, Fig. 1b). These four monomers allow four pairs of cationic and aromatic monomers. The two pairs of ATAC/PEA (R_1_ = R_2_ = H) and MATAC/PEMA (R_1_ = R_2_ = CH_3_) have the same vinyl heads, while the two pairs of ATAC/PEMA (R_1_ = H, R_2_ = CH_3_) and MATAC/PEA (R_1_ = CH_3_, R_2_ = H) have different vinyl heads.

**Fig. 2:**
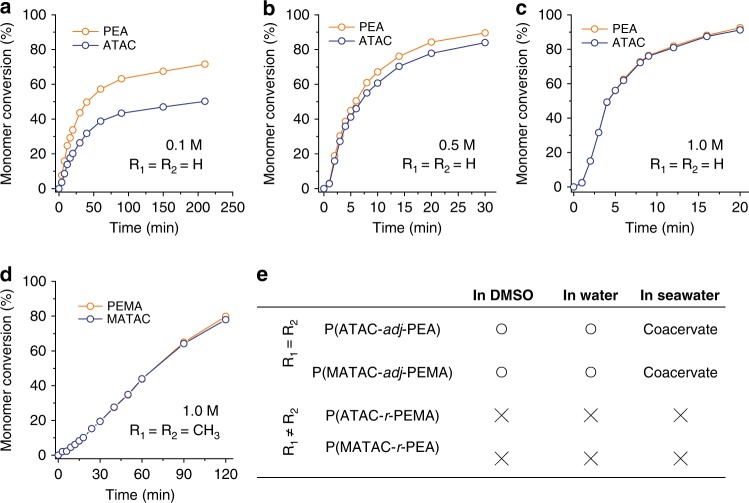
Monomer polymerization kinetics. **a**–**c** Monomer conversions for ATAC/PEA monomer pair (R_1_ = R_2_ = H) at different total molar concentrations. **d** Monomer conversions for MATAC/PEMA monomer pair (R_1_ = R_2_ = CH_3_) at 1.0 M. The monomer ratios of all pairs are 1:1. **e** Solubility of copolymers obtained in DMSO, water, and seawater. The circle and cross refer to “soluble” and “insoluble”, respectively

All the four pairs show the cation–π interactions in their common solvent, dimethyl sulfoxide (DMSO), and the interaction strength increases with the total monomer concentration, as revealed by nuclear magnetic resonance (NMR) spectroscopy at an equimolar ratio (Supplementary Fig. 1 and Supplementary Note 1). The conversion rates of the copolymers were studied by performing free-radical polymerization at equimolar ratio but different total monomer concentrations. For the two pairs having the same vinyl heads (R_1_ = R_2_), the cationic and aromatic monomers showed very different conversion rates at low concentrations (<1.0 M), but the difference decreased with the increase of total concentration, namely the strength of their cation–π interactions (Fig. 2a–d and Supplementary Fig. 2). Surprisingly, at a relatively high concentration (1.0 M), the conversion rates of the cationic and aromatic monomers were found to be the same throughout the reaction, indicating that the two monomers are equally incorporated into the copolymers. In contrast, for the two pairs with different vinyl heads (R_1_ ≠ R_2_), the cationic and aromatic monomers exhibited very different conversion rates even at high concentration (Supplementary Fig. 3), indicating the formation of copolymers containing cation- and aromatic-rich segments. It was further confirmed that the monomer pairs with the same vinyl heads but without cation–π interactions exhibit different monomer conversion rates (Supplementary Fig. 4). These results reveal that both the high concentration of the cationic–aromatic complexes in the reaction solution and the same chemical environment of the reactive vinyl heads are required for matched copolymerization.

It is hypothesized that the strong cation–π interaction may lead to the formation of a monomer pair complex that behaves like divinyl monomers or multi-vinyl monomers during the polymerization, which may dramatically increase the cross monomer reaction probability. However, such cross monomer reaction still requires the presence of same vinyl heads, because the cation–π interaction is a dynamic bond, different from the covalent divinyl monomer, which can overcome the inherently different reactivities of their methacrylate and acrylate units^21^. NMR measurement of poly(cation-*adj*-π) also confirmed that the cationic and aromatic residues were homogeneously dispersed in chains without any obvious one-component-rich segments (Supplementary Fig. 5, and Supplementary Note 2). Studies on the detailed reaction mechanism are in progress, and will be reported in future works.

Despite containing 50% hydrophobic aromatic monomers, the adjacent sequence of the monomers in poly(cation-*adj*-π) leads to good solubility of the copolymer both in DMSO and water (Fig. 2e and Supplementary Fig. 6). This contrasts with the poly(cation-*r*-π), which has cation- and aromatic-rich segments from R_1_ ≠ R_2_ monomer pairs that are insoluble both in DMSO and water. In the case of the adjacent cationic and aromatics sequences, the strong electrostatic repulsion of cationic residues prevents the hydrophobic aromatic residues from aggregating in polar solvents.

### Poly(cation-*adj*-π) in saline water

When the water-soluble poly(cation-*adj*-π) is dissolved in saline water (0.7 M, ionic strength of seawater), the polymer solution becomes turbid, indicating the formation of coacervates. This is so because salt ions screen long-range electrostatic repulsion and strengthen the effective cation–π and hydrophobic interactions between intra/inter chains (Fig. 2e and Supplementary Fig. 7, and Supplementary Note 3)^22^. Such strong cohesive interactions could be used for hydrogel formation.

Furthermore, the poly(cation-*adj*-π) coacervates exhibit strong adsorption on negatively charged SiO_2_ surface in 0.7 M NaCl solution, as revealed by a Quartz Crystal Microbalance (QCM) measurement (Supplementary Fig. 8). Compared to polycation P(ATAC), P(ATAC-*adj*-PEA) carrying only half concentration of cationic groups exhibited a much larger degree of adsorption on the SiO_2_ surface than P(ATAC), indicating that cation–π interactions can enhance the electrostatic interactions between ATAC and SiO_2_ surface in saline water.

### Gelation of poly(cation-*adj*-π) in saline water

**Fig. 3:**
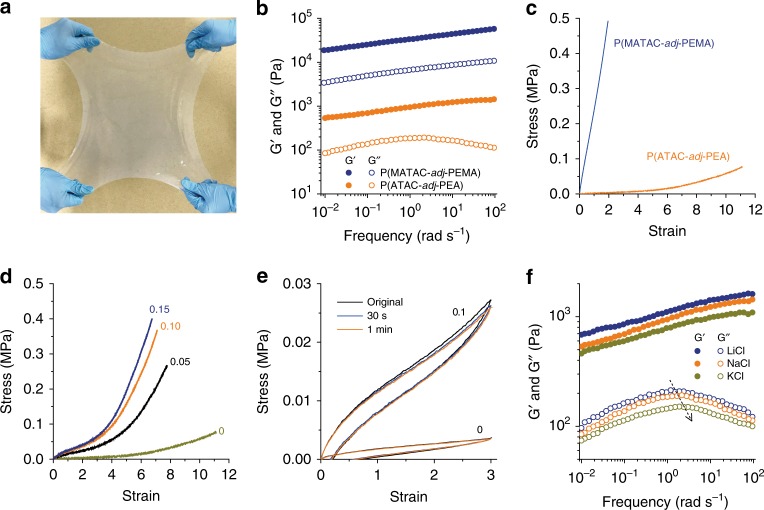
Mechanical properties of poly(cation-*adj*-π) gels. **a** The digital photo of a P(ATAC-*adj*-PEA)-0.1 hydrogel with a size of 235 mm × 235 mm × 6 mm. **b** Rheological behaviors of physical poly(cation-*adj*-π) gels in 0.7 M NaCl. **c** Tensile stress–strain curves of physical poly(cation-*adj*-π) gels in 0.7 M NaCl. **d** Tensile stress–strain curves of P(ATAC-*adj*-PEA) gels with different amounts of chemical crosslinkers. **e** Hysteresis and self-recovery of the physical P(ATAC-*adj*-PEA) gel and 0.1 mol% chemically crosslinked P(ATAC-*adj*-PEA)-0.1 gel measured by cyclic tensile tests. The initial strain rate of tensile tests was 0.14 s^−1^. The numbers in **d** and **e** are the chemical crosslinkers in mol% relative to monomers. **f** Rheological behaviors of physical P(ATAC-*adj*-PEA) gels in 0.7 M different alkali solutions

Based on the cohesion and adsorption abilities of poly(cation-*adj*-π) in saline water, we intend to develop adhesive hydrogels for the marine environment. The efficient one-pot free-radical polymerization allows the synthesis of poly(cation-*adj*-π) in a large quantity of hydrogels (Fig. 3a). Polymerization was performed at a high monomer concentration so that the resultant polymers are entangled, which prevents cohesion-induced macroscopic phase separation^23^. For instance, P(ATAC-*adj*-PEA) (R_1_ = R_2_ = H) synthesized at a total monomer concentration of *C*_m_ = 2.4 M substantially swells but does not dissolve in seawater-level saline solution (0.7 M NaCl aqueous solution) to form a physical hydrogel with a polymer content of 8 wt%, and P(MATAC-*adj*-PEMA) (R_1_ = R_2_ = CH_3_) synthesized at *C*_m_ = 2.2 M also forms a physical hydrogel but with a higher polymer content (27 wt%). The rheological tests reveal that these poly(cation-*adj*-π) gels are weakly viscoelastic (Fig. 3b), confirming the dynamic crosslinking mechanism of the network. The storage modulus (*G*′) of the P(MATAC-*adj*-PEMA) gel is ~40 times that of the P(ATAC-*adj*-PEA) gel, indicating that the hydrophobic methyl groups on vinyl group enhance the physical interactions between polymer chains. The relatively soft P(ATAC-*adj*-PEA) gel (Young’s modulus ~5 kPa) is highly stretchable (up to an elongation of 1,100%) compared to the relatively rigid P(MATAC-*adj*-PEMA) gel (Young’s modulus ~0.28 MPa, with elongation of 200%) (Fig. 3c). Adding a small amount of chemical crosslinker, *N*,*N*′-methylenebis(acrylamide) (MBAA), to the soft P(ATAC-*adj*-PEA) gels substantially enhances their mechanical strength (Fig. 3d). Moreover, the dynamic feature of physical interactions provides the hydrogels with energy dissipation and rapid self-recovery abilities. At a strain of 300%, distinct hysteresis is observed, indicating energy dissipation. The sample showed 100% recovery in the second loading after a 30-s wait (Fig. 3e).

To confirm that the cation–π interaction contributes to the dynamic crosslinking, the rheological behavior of P(ATAC-*adj*-PEA) gel was studied in various types of alkali salt solutions. In a previous study, it was revealed that the binding strength of cations to aromatic groups differs for cations, mainly depending on the hydration radius of the cation^24^. Cations with larger hydration radius initiate weaker cation–π interaction as they must expend a greater desolvation energy for the association. Because the poly(cation-*adj*-π) hydrogels swelled in the saline solution, besides the quaternary-N cations of the polymer, small cations of the simple salts can also bond with the aromatic groups, which competes with the quaternary-N/benzene cation–interactions. In aqueous solutions, the binding strength of cations to an aromatic group follows the order NR_4_^+^ > K^+^ > Na^+^ > Li^+^ (ref. ^25^). To study this influence, the hydrogels were also swelled in LiCl and KCl salt solutions (0.7 M), and their rheological behaviors were compared with NaCl (Fig. 3f). The storage modulus (G′) of the hydrogel in LiCl solution was stronger, whereas the one in KCl was slightly weaker than that in NaCl. Besides, the peak of loss modulus (G″) of the hydrogel slightly shifts to a lower angular frequency in LiCl and to a higher frequency in KCl. This is consistent with the fact that the quaternary-N/benzene cation–π interactions are slightly strengthened in LiCl, but weakened in KCl compared to that in NaCl because of competition with the small cations. These results in different alkali ions confirm that the cation–π interaction contributes to dynamic crosslinking.

### Adhesion of poly(cation-*adj*-π) gels in saline water

**Fig. 4:**
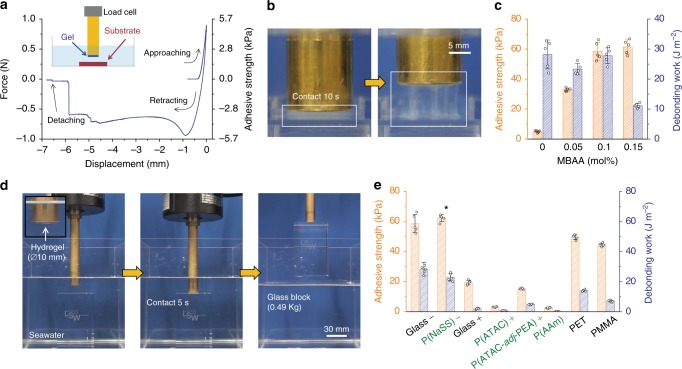
Adhesion of the poly(cation-*adj*-π) gels under seawater. **a** Force–displacement curves of the physical P(ATAC-*adj*-PEA) gel adhered to a negatively charged glass substrate in 0.7 M NaCl solutions. The insert is a schematic diagram of the tack test. **b** Photographs illustrating the bulk deformation of the physical P(ATAC-*adj*-PEA) gel during retraction from the glass substrate. The diameter and thickness of the gel were 15 mm and 1.6 mm, respectively. **c** Adhesion values of P(ATAC-*adj*-PEA) gels with different amounts of chemical crosslinkers MBAA to the glass substrates in 0.7 M NaCl solutions. **d** Photographs showing a P(ATAC-*adj*-PEA)-0.1 gel adhered to a 0.49-kg glass block in seawater and then lifted it up out of the seawater to the air. **e** Adhesion behaviors of P(ATAC-*adj*-PEA)-0.1 hydrogels to a variety of substrates with different types of surface charges and hydrophobicity in 0.7 M NaCl solution. The result marked by asterisk indicates that the fracture occurred in the bulk of the substrate hydrogel, but not at the interface. The error bars indicate the standard deviation (*n* = 5)

The dissipative poly(cation-*adj*-π) hydrogels act as excellent adhesives to negative surfaces in seawater. We tested the adhesiveness of poly(cation-*adj*-π) hydrogels to negatively charged glass substrates in 0.7 M saline water using a tack test (Supplementary Fig. 9)^7^. The soft physical P(ATAC-*adj*-PEA) hydrogel (polymer content ~8 wt%) adhered to the glass substrate strongly and underwent a large deformation during the retracting process before joint failure (Fig. 4a). The detachment occurred mostly at the interface but with some gel residue on the glass surface, indicating that the adhesion of the gel is comparable to its cohesion. Prior to debonding, a finger-like structure characteristic of strong adhesives was observed (Fig. 4b, and Supplementary Video 1).

The debonding work is high for the soft, stretchable physical P(ATAC-*adj*-PEA) gel, but its adhesive strength is quite low (Fig. 4c). By adding a small amount of chemical crosslinker, the debonding strength of hydrogels increased dramatically (Fig. 4c and Supplementary Fig. 10). The chemically crosslinked P(ATAC-*adj*-PEA)-0.1 gel (polymer content ~18 wt%) demonstrates strong adhesion to the glass substrate—the adhesive strength approached ~60 kPa with an adhesion energy of ~30 J m^−2^ (Fig. 4c). Owing to the expeditious formation of non-covalent electrostatic interactions at the interface, the adhesion is rapid and reversible. A contact dwelling time of 10 s at a pressure equivalent to the elastic modulus of the gel is enough for a saturated adhesive performance (Supplementary Fig. 11). The adhesion was reversible due to the noncovalent interactions at the interface, and the adhesion force was only slightly diminished over the many cycles of adhesion (Supplementary Fig. 12). Figure 4d demonstrates that the P(ATAC-*adj*-PEA)-0.1 gel with a 10-mm diameter and 1.24-mm thickness can rapidly adhere to a 0.49-kg glass block submerged in seawater, and the block can be lifted off of the seawater to air without failure of the adhesion (Supplementary Video 2).

Unlike catechol-based materials whose adhesiveness is readily weakened in seawater due to oxidization of the catechol group^26^, the functional groups, quaternary-N and phenyl, in the cation–π systems are insensitive to pH and oxygen. Therefore, the poly(cation-*adj*-π) hydrogels exhibited excellent adhesion in a wide range of pH of salt water, in addition to seawater (Supplementary Fig. 13). In addition, the adhesion strength of these hydrogels in 0.7 M NaCl solution did not decrease after 2 weeks, indicating that the adhesion has long-term stability (Supplementary Fig. 14). Furthermore, the proposed poly(cation-*adj*-π) hydrogels displayed good adhesiveness not only in the seawater-level ionic strength condition, but also over a wide range of concentration of NaCl solutions (0.1–1.0 M, Supplementary Fig. 15).

The adhesion of poly(cation-*adj*-π) hydrogels to diverse substrates were further tested (Fig. 4e). In a first, the hydrogels were found to exhibit excellent adhesion to negatively charged surfaces of both hard and soft materials. In theory, the electrostatic interaction between two oppositely charged surfaces should diminish in a high-salt solution due to the Debye screening effect^6^. However, the adhesions of P(ATAC-*adj*-PEA)-0.1 hydrogels to negatively charged substrates, such as a rigid glass surface or a soft P(NaSS) hydrogel, are very strong. In fact, the adhesions are so strong that the detachment occurred at the bulk parts of the soft P(NaSS) hydrogel. On the contrary, the adhesions to the positively charged substrates and neutral P(AAm) hydrogel were very weak. These results clearly reveal that the adhesion of poly(cation-*adj*-π) to negatively charged substrates has an electrostatic origin, although it diminishes for commonly charged hydrogels in such high ionic-strength environments (Supplementary Fig. 16). This result clearly demonstrates the importance of adjacent cationic and aromatic sequences to the effective electrostatic interaction in high ionic-strength environments.

Through the hydrophobic interaction, the poly(cation-*adj*-π) gels also showed relatively strong adhesion on hydrophobic solid surfaces, such as polyethylene terephthalate (PET) and polymethyl methacrylate (PMMA). In addition, the adhesion on the same P(ATAC-*adj*-PEA)-0.1 hydrogel (self-adhesion) is much stronger than that on the positive P(ATAC) hydrogel, indicating that self-adhesion is based on the cation–π interaction across the interface.

### Universality of the approach

**Fig. 5:**
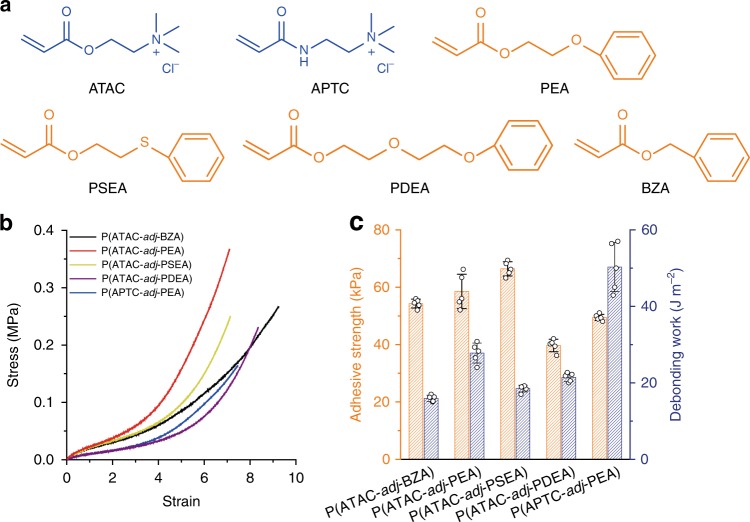
Universality of the strategy. **a** Chemical structures of other cationic and aromatic monomers, including ATAC, (3-acrylamidopropyl)trimethyl ammonium chloride (APTC), PEA, 2-(phenylsulfanyl)ethyl acrylate (PSEA), 2-(2-phenoxyethoxy)ethyl acrylate (PDEA), and benzyl acrylate (BZA). **b** Stress–strain curves of various poly(cation-*adj*-π) hydrogels. All the samples were prepared at equimolar ratio and with a 0.1-mol% chemical crosslinker. All the gels were equilibrated in 0.7 M NaCl solutions before the tests, and the initial strain rate was 0.14 s^−1^. **c** Adhesion values of various poly(cation-*adj*-π) hydrogels. The error bars indicate standard deviation (*n* = 5). The applied normal pressure was 100% of the elastic modulus of the gels for all the tests. All the tests were performed on a glass substrate in a 0.7 M NaCl solution

The rule that cationic and aromatic monomers with the same reactive vinyl heads (R_1_ = R_2_) form poly(cation-*adj*-π) having adjacent cationic and aromatic sequences is applicable to diverse combinations of monomers. More examples of poly(cation-*adj*-π) hydrogels (chemical crosslinker: 0.1 mol%) with different monomers are synthesized (Fig. 5a). All the combinations lead to viscoelastic, stretchable, and mechanically strong hydrogels (Fig. 5b). All poly(cation-*adj*-π) (R_1_ = R_2_ = H) exhibited strong adhesiveness to negatively charged glass in saline water (0.7 M, Fig. 5c). These results prove the universality of the approach discovered in this work.

The properties of poly(cation-*adj*-π)s strongly depend on the specific chemical structures of cationic and aromatic monomer pairs. For example, the chemically crosslinked P(MATAC-*adj*-PEMA)-0.1 hydrogel, from the monomer pair with R_1_ = R_2_ = CH_3_, exhibited very high modulus in 0.7 M salt solution due to strong dynamic bonds. The high modulus leads to weak adhesion (Supplementary Fig. 17). However, the linear P(MATAC-*adj*-PEMA)s can be used as an excellent glue in seawater. As an example, we test its gluing ability to sea sand that is negatively charged silicon dioxide. By mixing the viscous polymer/DMSO solution (23.6 wt%, 1.2 g) and sea sands (4 g), a sticky and flexible mixture could be produced (Supplementary Fig. 18). The mixture solidified into an elastic composite in seawater (Supplementary Fig. 19) because removal of DMSO enhances the adhesion of the polymer to sand. These results demonstrate that poly(cation-*adj*-π) has an immense potential as adhesives in marine applications. The diverse chemical structure of poly(cation-*adj*-π) gives a wide range of specific applications.

## Discussion

In this section, we discuss why adjacent cationic and aromatic sequences result in effective electrostatic interaction amid high ionic-strength environments. Previous studies have suggested that breaking down the hydration-layer barriers is the key step to obtaining underwater adhesion^27,28^. Nanomechanics measurements have shown that under an applied force, mussel foot proteins (mfps) can “clean” the surface, i.e., remove the bound salts and hydrophobic residues to dislodge the hydration-layer barriers, by the application of cationic lysine, paving the way for catechol adhesion^27,28^. This synergy promotes wet adhesion of mfps. In our system, the poly(cation-*adj*-π) in which cationic and aromatic monomers are adjacently located may show a similar effect with mfps by dehydrating the surface—under external compressive force, the cationic quaternary-N expels surface salt ions, while hydrophobic phenyl group breaks down the hydration layer. Consequently, the electrostatic interactions between the polymer and glass surfaces are established stably (Supplementary Fig. 20). Moreover, the hydrophobicity of aromatic residues may provide an interior region with low dielectric constant to enhance the electrostatic interactions^29^. During the retraction, the adjacent aromatic groups may play a vital role in preventing water molecules and salt ions from readily reinvading the interface, delaying the rehydration process. In addition to this strong interface bridging effect, the bulk energy dissipation of the viscoelastic gel also effectively delays the interfacial detachment, resulting in strong adhesiveness^30–32^; whereas, for the control samples without aromatic residues, or the non-adjacent cationic and aromatic residues, the expulsion of the hydration layer is hardly achievable, especially for hydrophilic polymers. Moreover, the electrostatic interactions at the interface are easily broken during the retraction process, leading to the weak adhesiveness of these hydrogels. Therefore, the adjacent location of phenyl and cationic quaternary-N contributes to the synergetic effect of strong adhesiveness.

Beyond the molecular interaction scale, the condensed coacervate structure, which has a high polymer density, low interfacial energy, etc., has been reported as being important to the enhancement of adhesion in sessile marine organisms^33–35^. Such supramolecular structures at a large scale may also play an important role in the strong adhesion in the proposed poly(cation-*adj*-π) hydrogels. In saline water, cation–π interaction leads to the complex coacervation of the copolymers. The condensed polymer chains provide multiple electrostatic interactions at the interface, which are more stable than a single interaction.

In mfps, the role of cationic residue is believed to work as vanguards to prepare the surface for its nearby catechol binding, while 3,4-dihydroxyphenyl-l-alanine (DOPA) is the key component for underwater adhesion^3,4,27,28^. However, in our study, the poly(cation-*adj*-π) hydrogels, free of DOPA residue, exhibit superior adhesiveness in seawater on a negatively charged surface, providing an alternative insight into the adhesion mechanism of adhesive proteins. In other words, cationic residues can also provide strong interfacial adhesion through electrostatic interactions to rock surfaces (negatively charged) even in a high ionic-strength environment, with the aid of the cation–π interactions with adjacent aromatic residues.

In summary, cation–π complex-aided copolymerization provides a route to the synthesis of sequence-controlled polymers by simple free-radical polymerization. The synthesized polymers with adjacent cationic–aromatic sequences not only provide the foundation for the development of adhesives working in saline water such as physiological and marine environments, but also give opportunities to study the electrostatic interactions in the high ionic-strength condition. This work may also enable researchers to re-recognize the importance of monomeric sequences to the properties of materials, which has been usually overlooked during material formation.

## Methods

### Synthesis of polymer hydrogels

The formulation of hydrogels is shown in Supplementary Table 1. All hydrogels were synthesized using the one-step random copolymerization of the prescribed monomers in DMSO. The monomers (molar ratio is 1:1) with the prescribed total monomer concentration (*C*_m_), 0.25-mol% UV initiator (2-oxoglutaric acid, relative to the total monomer molar concentration) and 0–0.15 mol% chemical crosslinker (MBAA, relative to the total monomer molar concentration) were first dissolved in DMSO, and then the resulting mixture was poured into a reaction cell consisting of a pair of glass plates with a 1-mm spacing and irradiated with a 365-nm UV light for 11 h. After the polymerization, the as-prepared gel was immersed in a large amount of 0.7 M NaCl (aqueous) solution to wash away the DMSO and residual chemicals. The saline water was exchanged every 12 h for over 1 week, after that the samples reached equilibrium. Before the test, the hydrogels were stored in 0.7 M NaCl solutions. The water content (*C*_W_) of hydrogels was measured using Moisture Balance (SHIMADZU, MOC-120H).

^1^H-NMR (Agilent 500 MHz) was performed to probe the cation–π interactions of the cationic and aromatic monomers in the DMSO solutions, and the monomer conversion for the free-radical polymerization. The tensile stress–strain measurements were performed using a universal testing machine (UTM, INSTRON 5965) at a steady velocity of 100 mm min^−1^ in air. Rheological tests were performed using an ARES-G2 rheometer (TA Instruments). The tack test was used to characterize the adhesiveness. The test was performed on the SHIMADZU tester (autograph AG-X) with Trapezium X software. To perform the experiment, the hydrogel with a diameter of 15 mm and thickness of ~1.2–2.0 mm was first glued to the probe using cyanoacrylate (super glue), and then the gel (on the probe) was immersed into the test solution for 5 min, so that it can reach equilibrium before the test. The probe approached the substrate surface at a speed of 10 μm s^−1^, held by the applied pressure for 10 s (value equals to the elastic modulus of the tested sample), and then retracted at a rate of 100 μm s^−1^. Except stated otherwise, all the tests were performed under 0.7 M NaCl solution. Further details on the methods are available in the Supplementary Information.

## Supplementary information

Supplementary Information

Description of Additional Supplementary Files

Supplementary Movie 1

Supplementary Movie 2

## Data Availability

Authors can confirm that all relevant data are included in the paper and/or its supplementary information files.
