# Supplementary material for: Adjacent cationic–aromatic sequences yield strong electrostatic adhesion of hydrogels in seawater
Source: Nat Commun. 2019 Nov 12;10:5127. doi: 10.1038/s41467-019-13171-9 (PMC6851134; doi:10.1038/s41467-019-13171-9)
Supplement: Supplementary file 1 — Supplementary Information [file 41467_2019_13171_MOESM1_ESM.pdf]

# Supplementary Information for

## Adjacent cationic–aromatic sequences yield strong electrostatic adhesion of hydrogels in seawater

Hailong Fan[1,2], Jiahui Wang[3], Zhen Tao[3], Junchao Huang[2], Ping Rao[3], Takayuki Kurokawa[2,4], and Jian Ping Gong[1,2,4*]

[1]Institute for Chemical Reaction Design and Discovery (WPI-ICReDD), Hokkaido University, N21W10, Kita-ku, Sapporo 001-0021, Japan

[2]Faculty of Advanced Life Science, Hokkaido University, N21W11, Kita-ku, Sapporo 001-0021, Japan

[3]Graduate School of Life Science, Hokkaido University, N21W11, Kita-ku, Sapporo 001-0021, Japan

[4]Global Station for Soft Matter GI-CoRE, Hokkaido University, N21W11, Kita-ku, Sapporo 001-0021, Japan

*Correspondence to: gong@sci.hokudai.ac.jp

## Supplementary Methods

### Materials

2-(acryloyloxy)ethyl trimethyl ammonium chloride (ATAC, 79.4% in water), 2-phenoxyethyl acrylate (PEA), benzyl acrylate (BZA), 2-(2-phenoxyethoxy)ethyl acrylate (PDEA), 2-(phenylsulfanyl)ethyl acrylate (PSEA), and 2-methoxyethyl acrylate (MEA) were provided by Osaka Organic Chemical Co., Ltd., Japan. Sodium p-styrenesulfonate (NaSS), N,N'-methylenebis(acrylamide) (MBAA), 2-oxoglutaric acid, salts (LiCl, NaCl, KCl), and dimethyl sulfoxide (DMSO) were purchased from Wako Pure Chemical Industries, Ltd. (3-acrylamidopropyl)trimethylammonium chloride (APTC, 74~76% in water), 2-(methacryloyloxy)ethyl trimethylammonium chloride (MATAC, ~80% in water), and 2-phenoxyethyl methacrylate (PEMA, purity > 85%) were purchased from Tokyo Chemical Industry Co., Ltd. 2-(dimethylamino)ethyl acrylate (DMAEA, 80% in water) was purchased from Sigma-Aldrich. All of the chemicals were used as purchased without further purification. Millipore deionized water was used in all of the experiments. The seawater was taken from coastal waters of Ishikari city, Hokkaido, Japan. Suspended solids in the seawater were removed by membrane filters (OMNIPORE$^{TM}$, 0.45 μm) before use.

### Characterization of polymerization kinetics

The monomer conversion for the free radical polymerization in DMSO was analyzed using with $^1$H-NMR reaction kinetics study. The mixed solution with different total monomer concentration of 1:1 monomer ratio and 0.25 mol% UV initiator (2-oxoglutaric acid, in a concentration relative to the total monomer concentration) in DMSO were polymerized in the glass vials under the irradiation of UV light (3.9 mW cm$^{-2}$) in the glove box. To study the monomer conversion during the copolymerization, 150 μL of the sample was taken from the reaction sample tube at different reaction times and transferred from the glove box to air immediately to quench the reaction, and then added into 300 μL DMSO-$d_6$ solution. The concentration of unreacted monomers remaining in solution was determined from the integral area ratio of $^1$H-NMR signals.

### Synthesis of polymer hydrogels

The formulation of hydrogels is shown in Supplementary Table 1. All hydrogels were synthesized using the one-step random copolymerization of the prescribed monomers in DMSO. The monomers (molar ratio is 1:1) with the prescribed total monomer concentration ($C_m$), 0.25-mol% UV initiator (2-oxoglutaric acid, relative to the total monomer molar concentration) and 0–0.15 mol%

chemical crosslinker (MBAA, relative to the total monomer molar concentration) were first dissolved in DMSO, and then the resulting mixture was poured into a reaction cell consisting of a pair of glass plates with a 1-mm spacing and irradiated with a 365-nm UV light for 11 h. After the polymerization, the as-prepared gel was immersed in a large amount of 0.7 M NaCl (aqueous) solution to wash away the DMSO and residual chemicals. The saline water was exchanged every 12 h for over 1 week, after that the samples reached equilibrium. Before the test, the hydrogels were stored in 0.7 M NaCl solutions.

The water content ($C_W$) of hydrogels was measured using Moisture Balance (SHIMADZU, MOC-120H). We assumed that the salt concentration ($C_{salt}$) in the gel is equal to that in the outer solution. The polymer content ($C_P$) was calculated from $C_W$ as:

$$C_p = 100\% - C_w - C_w \times C_{salt} \times M_{salt}/\rho_{H2O}$$

where $\rho_{H2O}$ is the density of water, $C_{salt}$ is the molar concentration of salt in water, and $M_{salt}$ is the molar weight of salt.

Tensile test

The tensile stress–strain measurements were performed using a universal testing machine (UTM, INSTRON 5965) at a steady velocity of 100 mm $min^{-1}$ in air. The samples were cut into a dumbbell-shape with the standard JIS-K6251-7 size (12 mm (L) × 2 mm (d) × 1~2 mm (w)). The elastic modulus ($E$) was calculated from the slope over 4–8% of the strain of the stress–strain curve. The tensile stress was calculated from the tensile force divided by the cross-section area of the virgin sample. The tensile strain was calculated from the displacement of cross-head of the testing machine divided by the initial gauge length (12 mm). The initial strain rate was calculated from the deformation velocity divided by the initial gauge length.

**Rheological test**

Rheological tests were performed using an ARES-G2 rheometer (TA Instruments). A rheological angular frequency sweep from 0.01 to 100 rad $s^{-1}$ was performed with a shear strain of 0.1% in the parallel-plate geometry at 24°C. The disc-shaped samples with thicknesses of approximately 1.5 mm and diameters of 15 mm were placed on the plates and surrounded by 0.7 M NaCl saline solution.

**Static polymer absorption on $SiO_2$**

The amount of polymer adsorbed on $SiO_2$ surface was measured by Quartz Crystal Microbalance (QCM, AFFINIX QN Pro, QCM2008-PRKIT, with frequency 27 MHz). The $SiO_2$-

coated sensor was first washed in UV. The 450-μL 0.7 M NaCl solution was added into the chamber and left undisturbed until the frequency stabilized. After that, a 4.5-μL polymer solution (10 mg $mL^{-1}$ in 0.7 M NaCl) was added into the chamber and left undisturbed until equilibrium (final polymer concentration 0.1 mg $mL^{-1}$).

### Measurement of adhesiveness

The tack test was used to characterize the adhesiveness. The hard substrates used were commercially available glass (Matsunami Glass, Osaka, Japan, S2112), positively charged glass (MAS-coated Superfrost, Matsunami Glass, Osaka, Japan, S9441), polyethylene terephthalate (PET), and polymethyl methacrylate (PMMA). The hydrogels, P(NaSS) gel, P(ATAC-*adj*-PEA)-0.1 gel, and P(AAm) gel were used as soft substrates (see synthesis method below). The hard substrates were rinsed with ethanol, and then with deionized water before use. The soft substrates were swollen in the 0.7 M NaCl saline water to reach equilibrium before use.

The test was performed on the SHIMADZU tester (autograph AG-X) with Trapezium X software. To perform the experiment, the hydrogel with a diameter of 15 mm and thickness of approximately 1.2–2.0 mm was first glued to the probe using cyanoacrylate (super glue), and then the gel (on the probe) was immersed into the test solution for 5 min, so that it can reach equilibrium before the test. The probe approached the substrate surface at a speed of 10 μm $s^{-1}$, held by the applied pressure for 10 s (value equals to the elastic modulus of the tested sample), and then retracted at a rate of 100 μm $s^{-1}$. Except stated otherwise, all the tests were performed under 0.7 M NaCl solution.

### Synthesis of soft substrates for adhesion test

All hydrogels were synthesized by polymerization of a 10-mL mixed solution with the prescribed chemicals (Supplementary Table 2) in a reaction cell with 1-mm spacing under UV light for 11 h. After polymerization, the as-prepared gels were immersed in a large amount of 0.7 M NaCl solution to wash the residual chemicals. Before the test, the hydrogels were stored in 0.7 M NaCl solutions. The thicknesses of these hydrogels were in the range of 1.5–2.0 mm.

**Supplementary Note 1: Characterization of cation–π interaction of monomers**

$^1$H-NMR (Agilent 500 MHz) was performed to probe the cation–π interactions of the cationic and aromatic monomers in the DMSO solutions. The two monomers were dissolved in DMSO-$d_6$ solution with different total monomer concentrations (0.1 M, 0.5 M, 0.7 M, 1.0 M, 1.5 M, and 2.0 M) at a fixed monomer ratio of 1:1, to conduct the NMR. The peak of the chemical shift of protons on DMSO was set as the reference in all systems. Supplementary Figure 1a and 1c show the typical $^1$H-NMR spectra of ATAC/PEA and MATAC/PEMA mixtures, respectively. With the change in the monomer concentration, the peaks of chemical shifts of protons on an aromatic ring (g, f) shift to low values (Supplementary Figure 1b and 1d), which indicate that the chemical environments of the phenyl group in the mixtures change in the presence of cationic monomers. As for control, the pair of a neutral DMAEA monomer and PEA (Supplementary Figure 1e), the protons on the aromatic ring remained unchanged in mixtures with different monomer concentrations (Supplementary Figure 1f). The chemical shifts of proton *g* on phenyl groups for all four different cationic and aromatic pairs, as well as a neutral/aromatic pair are plotted in Supplementary Figure 1g. By changing the total monomer concentration, the peak of protons on the aromatic ring shifts for all the four cation–π systems, but not for the neutral–π system. The results imply that the changes in chemical shift observed for phenyl protons in cationic/aromatic monomer mixtures are caused by the cation–π interactions.

**Supplementary Note 2: Characterization of monomer sequence of copolymers**

The sequence of aromatic residues on copolymer chains was characterized by $^1$H-NMR. Supplementary Figure 5a shows the partial (aromatic protons) $^1$H-NMR spectra of P(ATAC-*co*-PEA) with different PEA fractions, f, in DMSO. Firstly, we compare the NMR spectra of PEA monomer and homopolymer poly(PEA) (f 1.0). The aromatic proton signals of poly(PEA) shift to the higher field and broaden due to different chemical environments on the polymer chains. For copolymers, when the fraction of PEA is low ($f \leq 0.5$), the aromatic signals broaden slightly, but maintain almost the same position with the monomers, indicating no apparent aromatic-rich sequence in the copolymer. However, for copolymers bearing high PEA molar fraction ($f > 0.5$), the signals show a broad shoulder at the higher field (indicated by arrows), and the shoulders shift to the higher chemical field by increasing the PEA fraction, indicating the formation of aromatic-rich segments in the copolymer chains. This broad shoulder at higher field is a signature to identify the existence of aromatic-rich segment in copolymers.

Accordingly, we compared the NMR signals of copolymers from four pairs of monomers (monomer ratio 1:1). Supplementary Figure 5b shows the partial (aromatic protons) $^{1}$H-NMR spectra of copolymers in DMSO. For copolymers from a pair of monomers having the same vinyl group (P(ATAC-*adj*-PEA) and P(MATAC-*adj*-PEMA)), the signals show symmetric broadening around the peak of phenyl protons of the aromatic monomer, but no obvious shift. However, the signal of the other two copolymers (P(ATAC-*r*-PEMA) and P(MATAC-*r*-PEA)) have broad shoulders at the higher field (indicated by arrows). The NMR results shown above indicate that at the equimolar ratio, the pairs of cationic and aromatic monomers having the same vinyl groups ($R_1 = R_2$) can form a copolymer with adjacently dispersed cationic and aromatic residues. Otherwise ($R_1 \neq R_2$), the obtained copolymers have inhomogeneously dispersed residues containing both cationic-rich and aromatic-rich segments.

**Supplementary Note 3: Characterization of cation–π interaction of copolymers**

Poly(cation-*adj*-π) is soluble in water because the strong electrostatic repulsion of cationic residues prevent the adjacent hydrophobic residues from aggregating; in salt water (0.7 M NaCl), the salt ions screen the long-range electrostatic repulsion and strengthen the effective attraction between cationic and aromatic groups, forming strong intra-chain/inter-chain cation–π interactions, which causes the formation of coacervate[1]. Raman spectroscopy (RENISHAW, inVia Reflex, with a 532-nm laser light as an excitation source and a power of 10 mW) was performed to probe cation–π interactions in the system. In the Raman spectra, the ring breathing mode, centered at 998 $cm^{-1}$, appears as a very strong band compared to the adjacent 1,028 $cm^{-1}$ band (out-of-plane bending of

C-$H_{ring}$) of polymer in water. Upon the addition of salts, the intensity ratio, $I_{998}/I_{1028}$, changed from 2.92 in water to 2.19 in saline water (Supplementary Figure 7), indicating the strengthening of cation–π interactions in saline solution. The reduction of intensity of the ring breathing mode observed in the systems is a result of the formation of cation–π interactions between quaternary-N in the cationic monomers and phenyl group in the aromatic monomers, because an interaction of this type reduces the polarizability of the π-electron density in the ring[2].

## Supplementary Figures

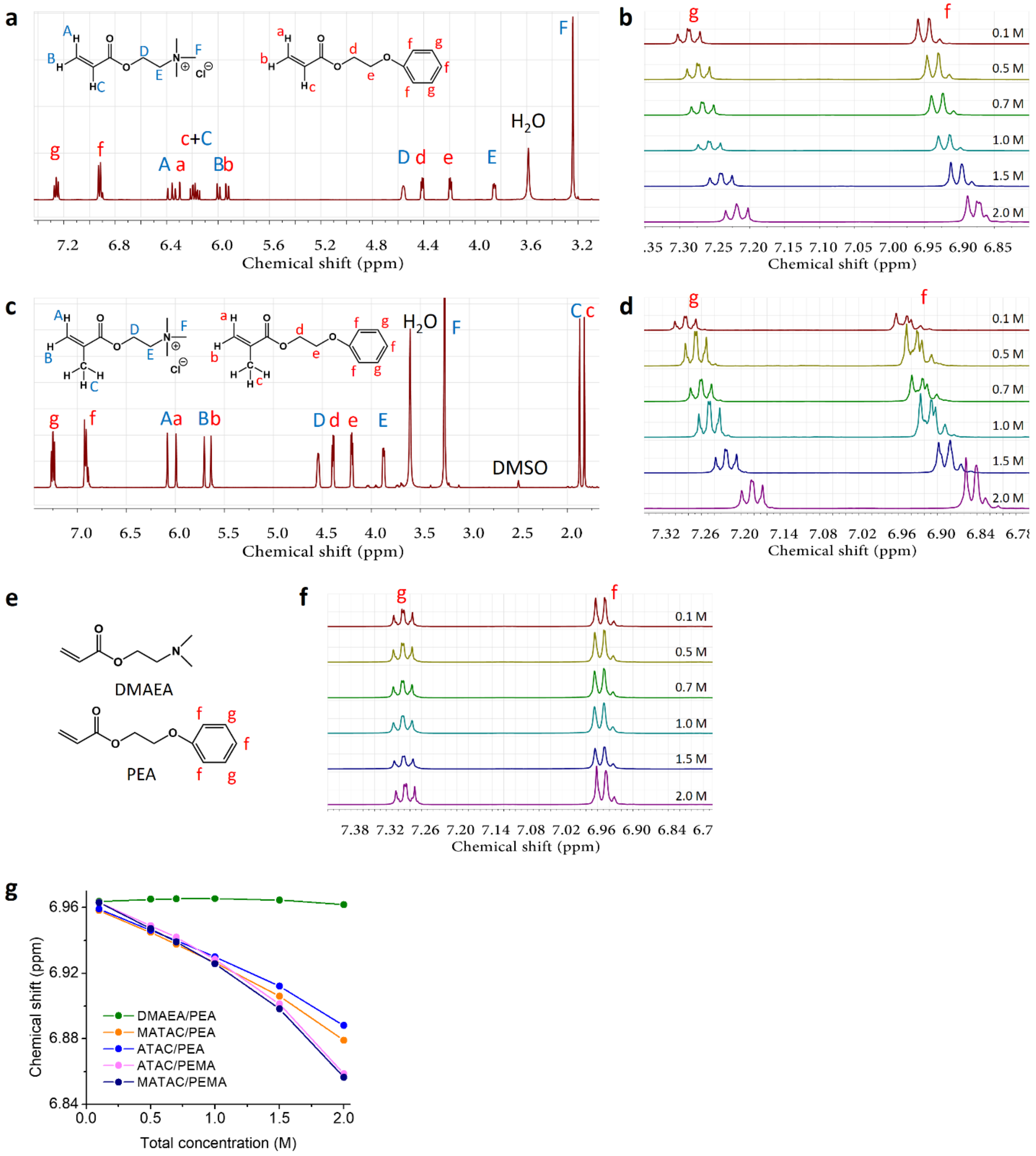


**Supplementary Figure 1. Characterization of cation–π interactions between a quaternary-N group of cationic monomers and phenyl group of aromatic monomers in DMSO. a,** $^1$H-NMR spectra of ATAC and PEA dissolved in DMSO-$d_6$, at a total monomer concentration of 1.0 M. **b,** Partial (aromatic protons) $^1$H-NMR spectra of ATAC/PEA mixtures with different total monomer concentrations. **c,** $^1$H-NMR spectra of MATAC and PEMA dissolved in DMSO-$d_6$, at a total

monomer concentration of 1.0 M. **d,** Partial (aromatic protons) $^{1}$H-NMR spectra of MATAC/PEMA mixtures with different total monomer concentrations. **e,** Chemical structures of neutral monomers DMAEA and PEA. **f,** Partial $^{1}$H-NMR spectra of DMAEA/PEA mixtures with different total monomer concentrations. **g,** The chemical shifts of the proton on phenyl ring (g) for five pairs plotted against total monomer concentrations. The monomer ratios in all systems are 1:1.

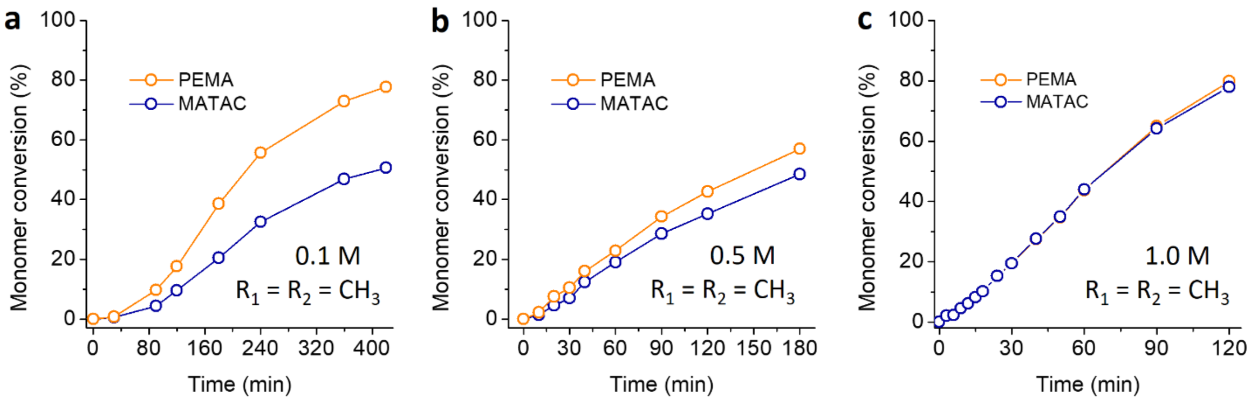


**Supplementary Figure 2.** Monomer conversions for MATAC/PEMA monomer pair ($R_1 = R_2 = CH_3$) at different total molar concentrations. The monomer ratio of all pairs is 1:1.

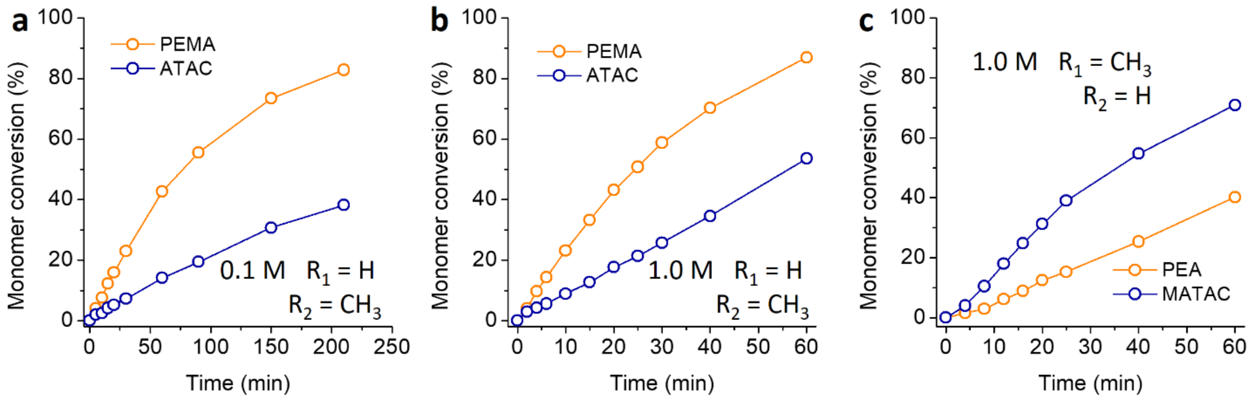


**Supplementary Figure 3.** Monomer conversions for $R_1 \neq R_2$ monomer pairs, ATAC/PEMA at different total molar concentrations **a, b**, and MATAC/PEA at 1.0 M **c**. The monomer ratio of all pairs is 1:1.

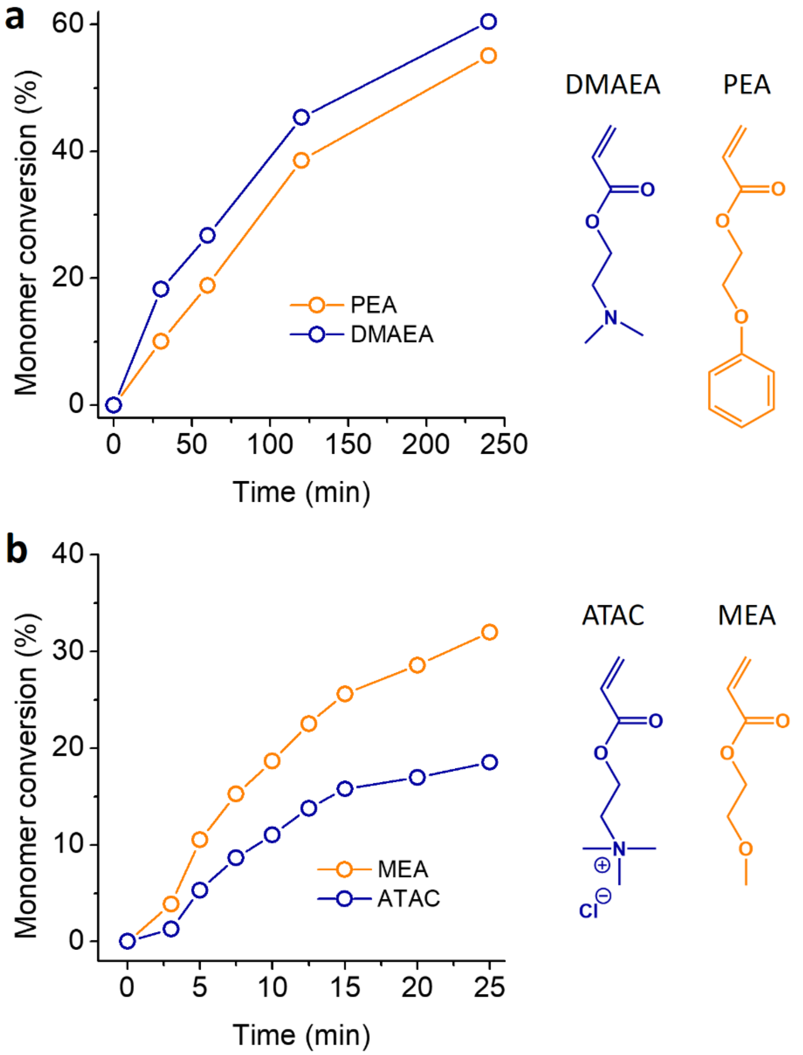


**Supplementary Figure 4.** Reaction time evolution of monomer conversions of monomer pairs with same vinyl heads but no cation–π interactions. **a,** Neutral/aromatic pair DMAEA/PEA system. **b,** Cationic/neutral pair ATAC/MEA system. The monomer ratio of all systems is 1:1, and the total monomer concentration is 1.0 M.

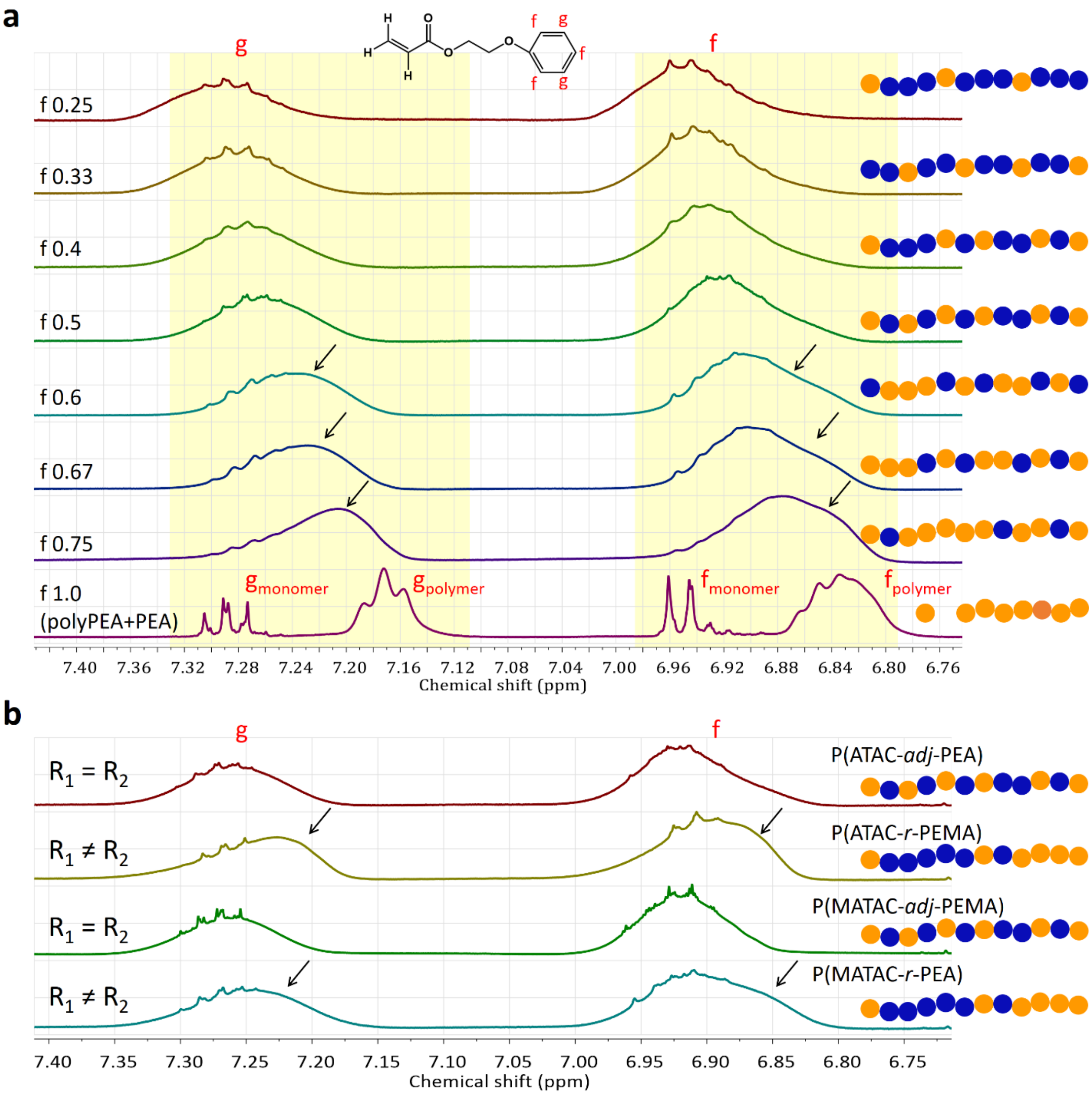


**Supplementary Figure 5. a,** Partial (aromatic protons) $^1$H-NMR spectra of P(ATAC-*co*-PEA) with different PEA fractions. **b,** Partial (aromatic protons) $^1$H-NMR spectra of copolymers from different pairs of monomers. For the scheme of polymer chains shown on the right, the blue and orange dots indicate cationic and aromatic monomers, respectively.

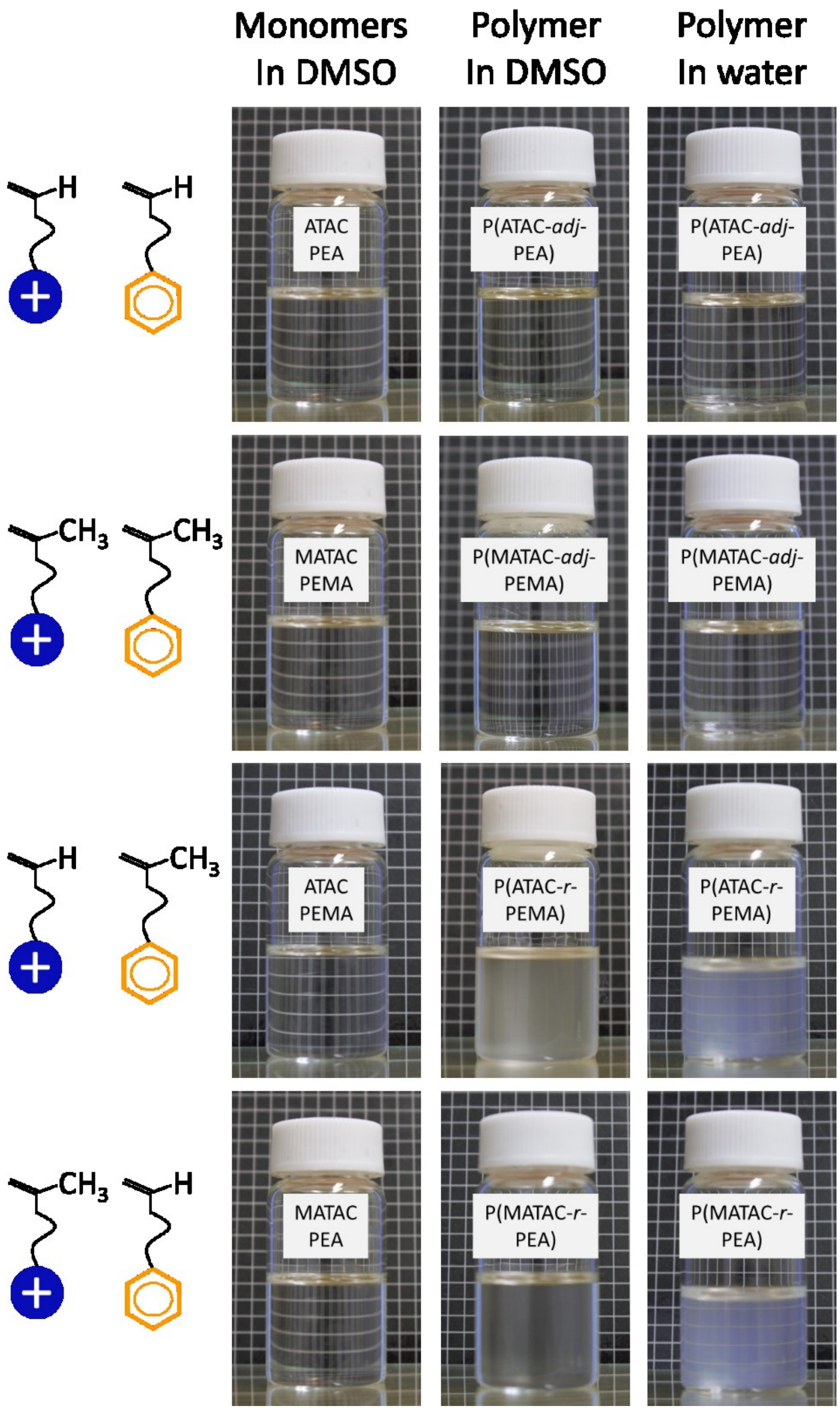


**Supplementary Figure 6.** Digital photographs of monomer solutions and their respective copolymer solutions in DMSO and water for various cationic and aromatic monomer pairs.

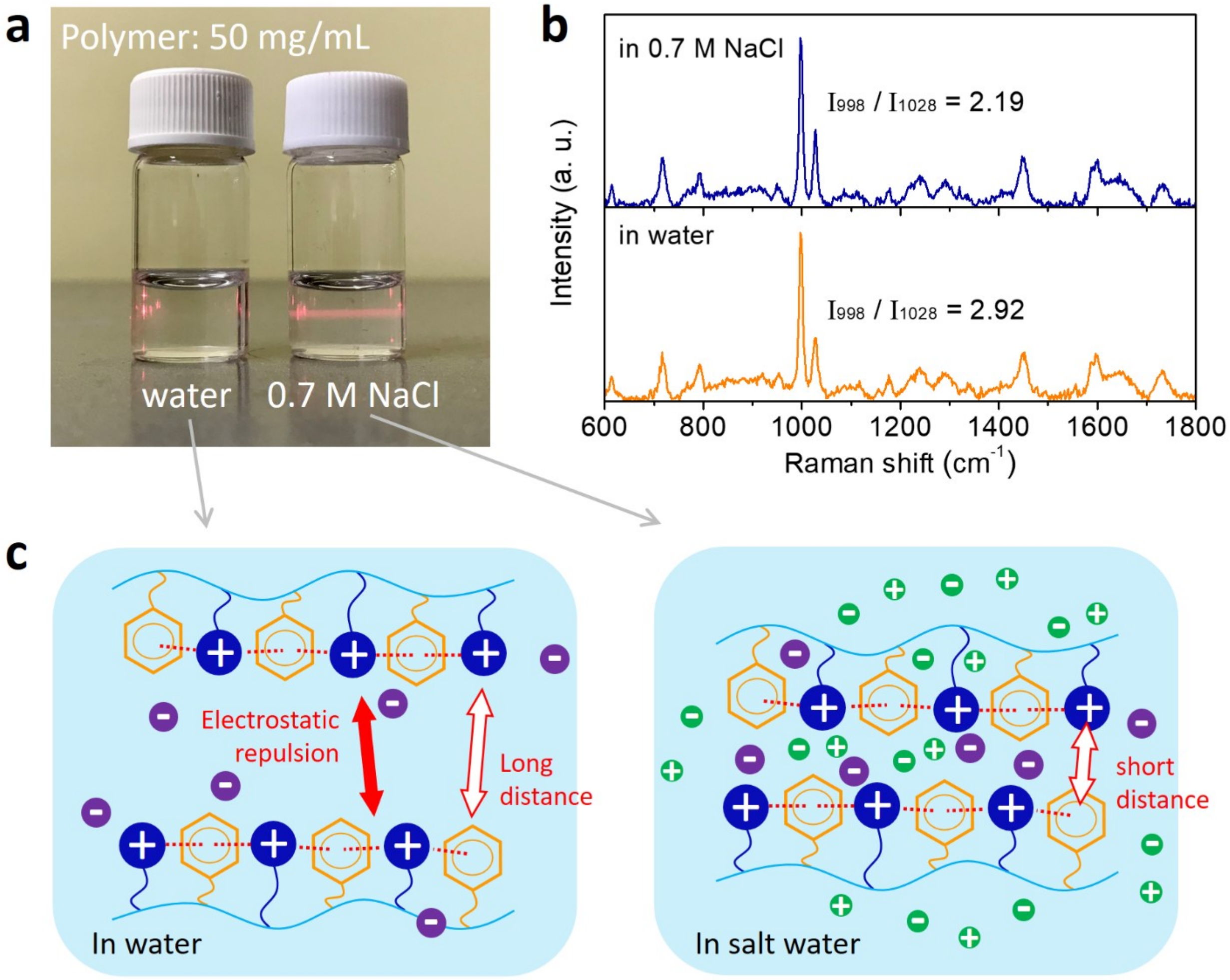


**Supplementary Figure 7. Characterization of cation–π interaction of copolymers. Cation–π interactions between quaternary-N and phenyl group in P(ATAC-*adj*-PEA) polymer. a,** The photographs of P(ATAC-*adj*-PEA) polymer dissolved in water and in 0.7 M NaCl solution exposed to a laser. The sample was transparent in water and became turbid in NaCl solution due to coacervation. **b,** Raman spectra of P(ATAC-*adj*-PEA) polymer dissolved in 0.7 M NaCl solution and water. **c,** Schematic illustration of the formation of coacervate in salt water.

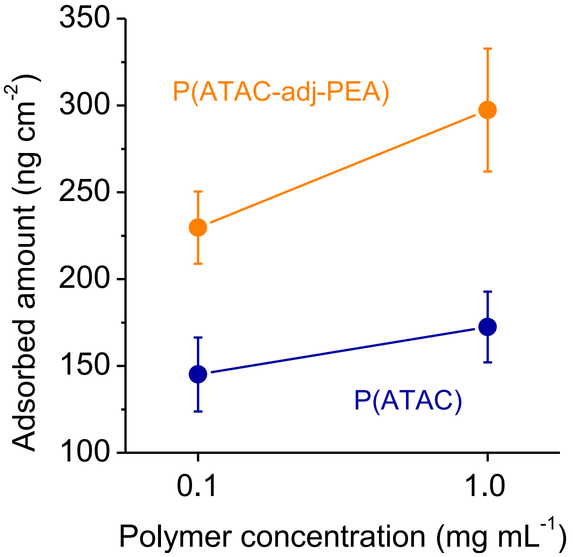


**Supplementary Figure 8.** Static polymer adsorption on $SiO_2$ surface measured by QCM in 0.7 M NaCl solution.

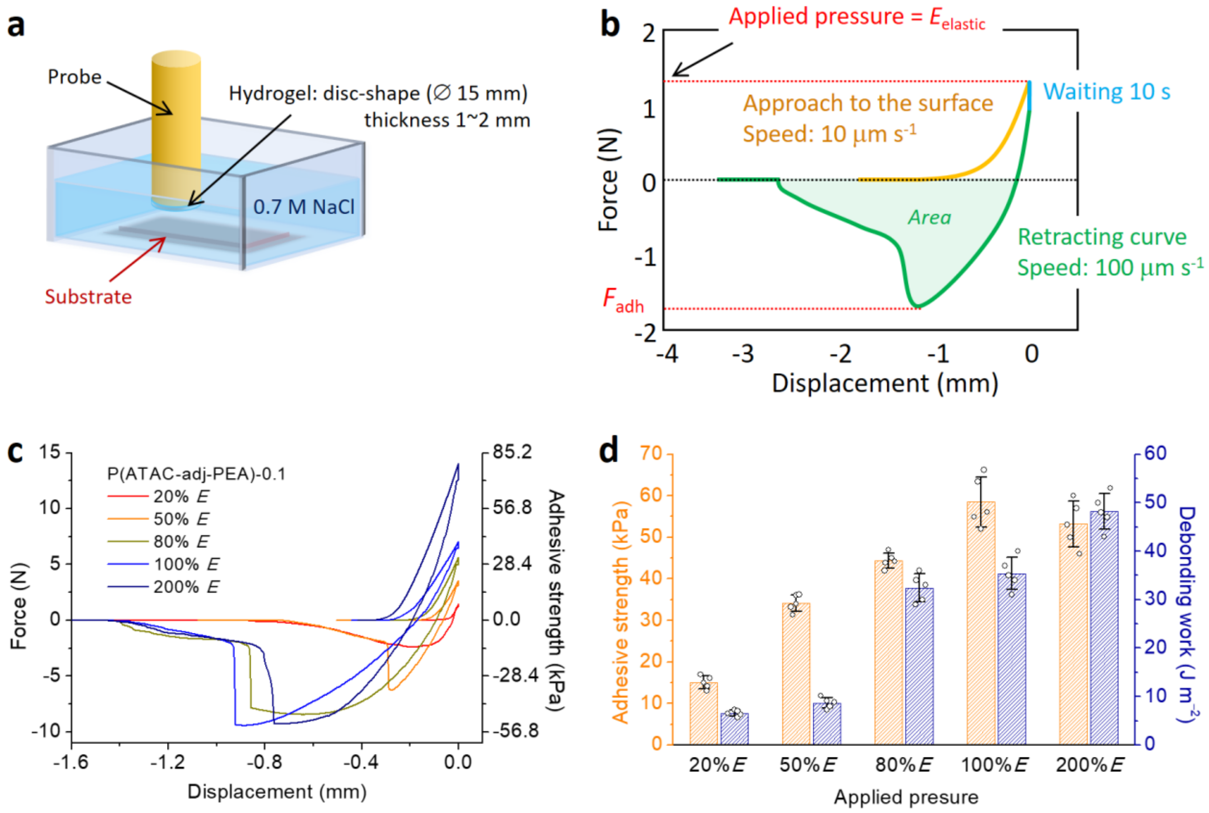


**Supplementary Figure 9. Adhesion measurement in this study. a,** Schematic diagram of tack test. **b,** Illustration of the force–displacement curve with corresponding test parameters. The Debonding work was calculated by Work Area divide by Contact Area. **c,** The measured force–displacement curves of P(ATAC-*adj*-PEA)-0.1 gels adhesion to the glass substrates under various applied pressures in terms of the gel's elastic modulus, *E*. **d,** Adhesion values of P(ATAC-*adj*-PEA)-0.1 gels under various applied pressures. The error bars indicate SD (N = 5). The test results reveal that the value of the optimized applied pressure is equal to the elastic modulus of the corresponding hydrogel.

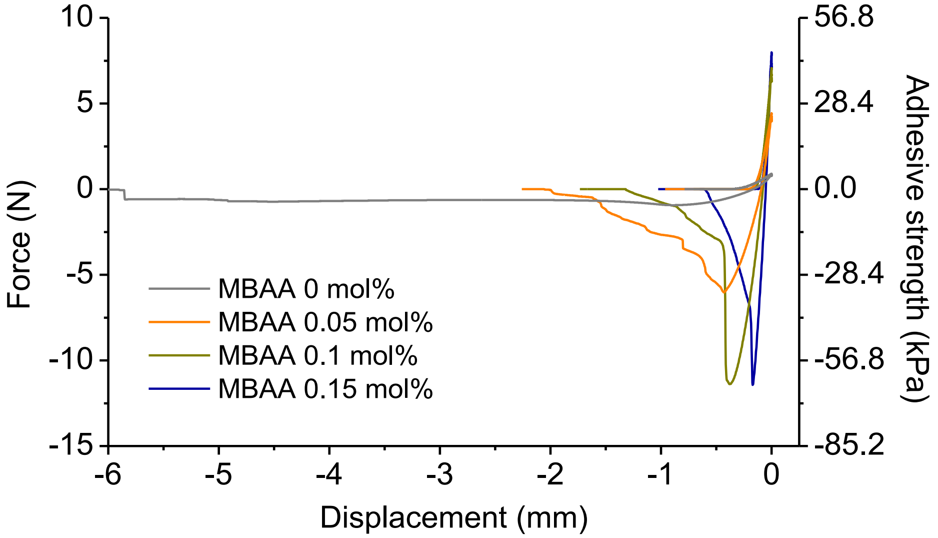


**Supplementary Figure 10.** Force–displacement curves of P(ATAC-*adj*-PEA) gels with different amounts of chemical crosslinkers adhering to the glass substrates in 0.7 M NaCl solutions. All samples were equilibrated in 0.7 M NaCl solutions. The tack tests were performed at a normal pressure equivalent to the elastic modulus *E* of the gels.

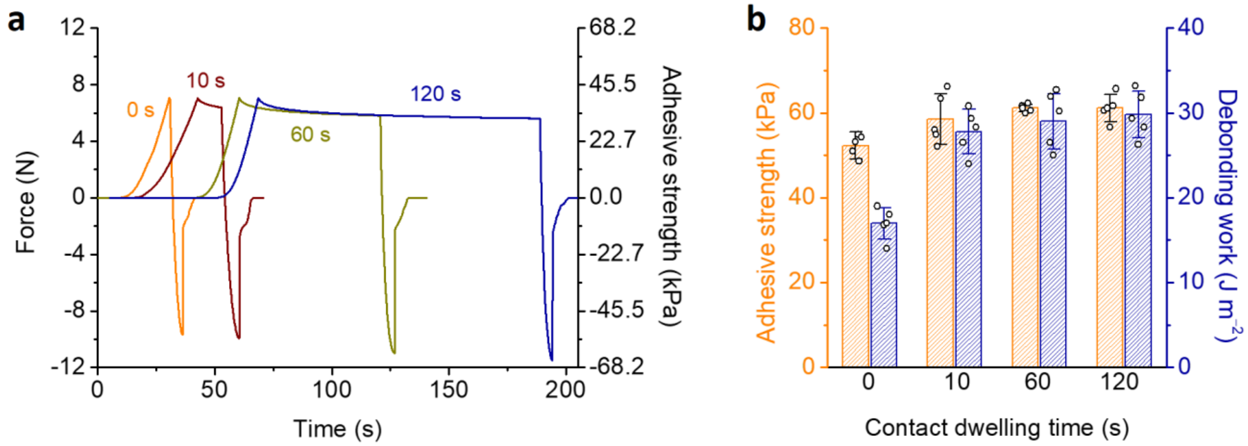


**Supplementary Figure 11.** Adhesion abilities of P(ATAC-*adj*-PEA)-0.1 gels at different contact dwelling times (0, 10, 60, and 120 s, respectively). **a,** Force–time curves. **b,** Adhesion values of gels. Error bars indicate SD (N = 5). The tack tests were performed at a normal pressure equivalent to the elastic modulus *E* of the gels. All the tests were performed on a glass substrate in a 0.7 M NaCl solution.

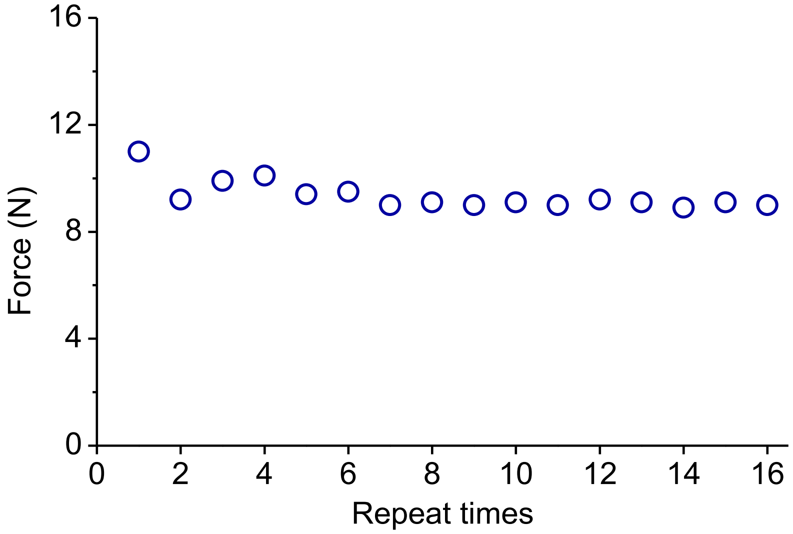


**Supplementary Figure 12.** Repeated adhesion of the P(ATAC-*adj*-PEA)-0.1 gel. The sample was placed at rest underwater for 5 min between two successive tests, and a new glass substrate was used for each test. The normal pressure applied was 100% of the elastic modulus of the gels for all the tests. All the tests were performed on a glass substrate in a 0.7 M NaCl solution.

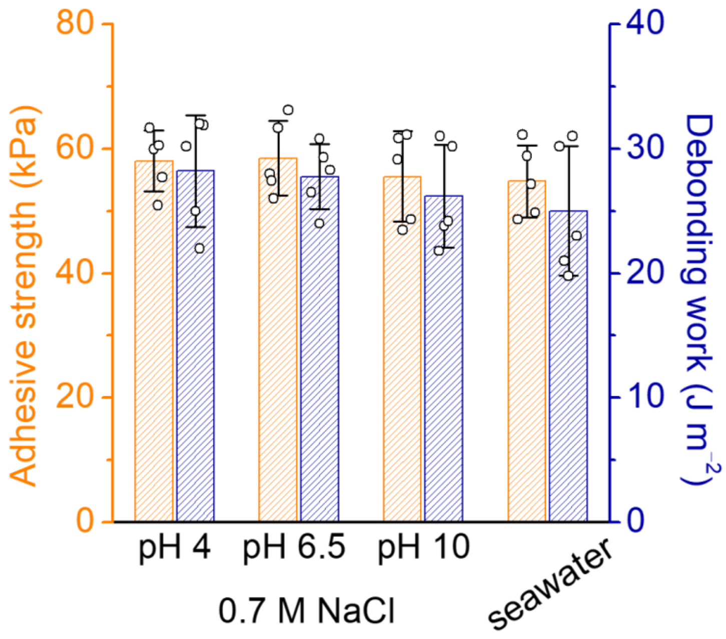


**Supplementary Figure 13.** Adhesion values of the P(ATAC-*adj*-PEA)-0.1 gel under 0.7 M NaCl solutions of different pH values and seawater measured at a dwelling time of 10 s. Error bars indicate standard deviation (N = 5). The tack tests were performed at a normal pressure equivalent to the elastic modulus $E$ of the gels. All the tests were performed on a glass substrate.

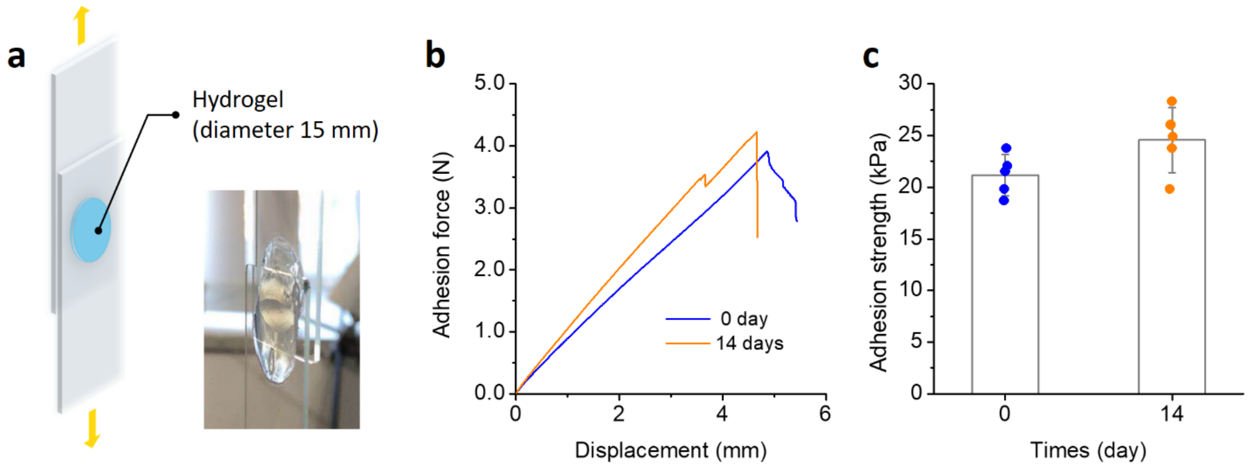


**Supplementary Figure 14.** (a) Schematic diagram of the lap shear test to measure the adhesion. The adhered plates were clamped to the universal testing machine and then were pulled at a crosshead speed of 10 mm min$^{-1}$. The adhesion strength was calculated by the measured maximum load divided by the bonded area. Each sample was tested three times in parallel. (b) The measured force–displacement curves of P(ATAC-*adj*-PEA)-0.1 gels adhesion to the glass substrates before and after soaking in 0.7 M NaCl solution. (c) Adhesion strength of P(ATAC-*adj*-PEA)-0.1 gels. Error bars indicate standard deviation (N = 5).

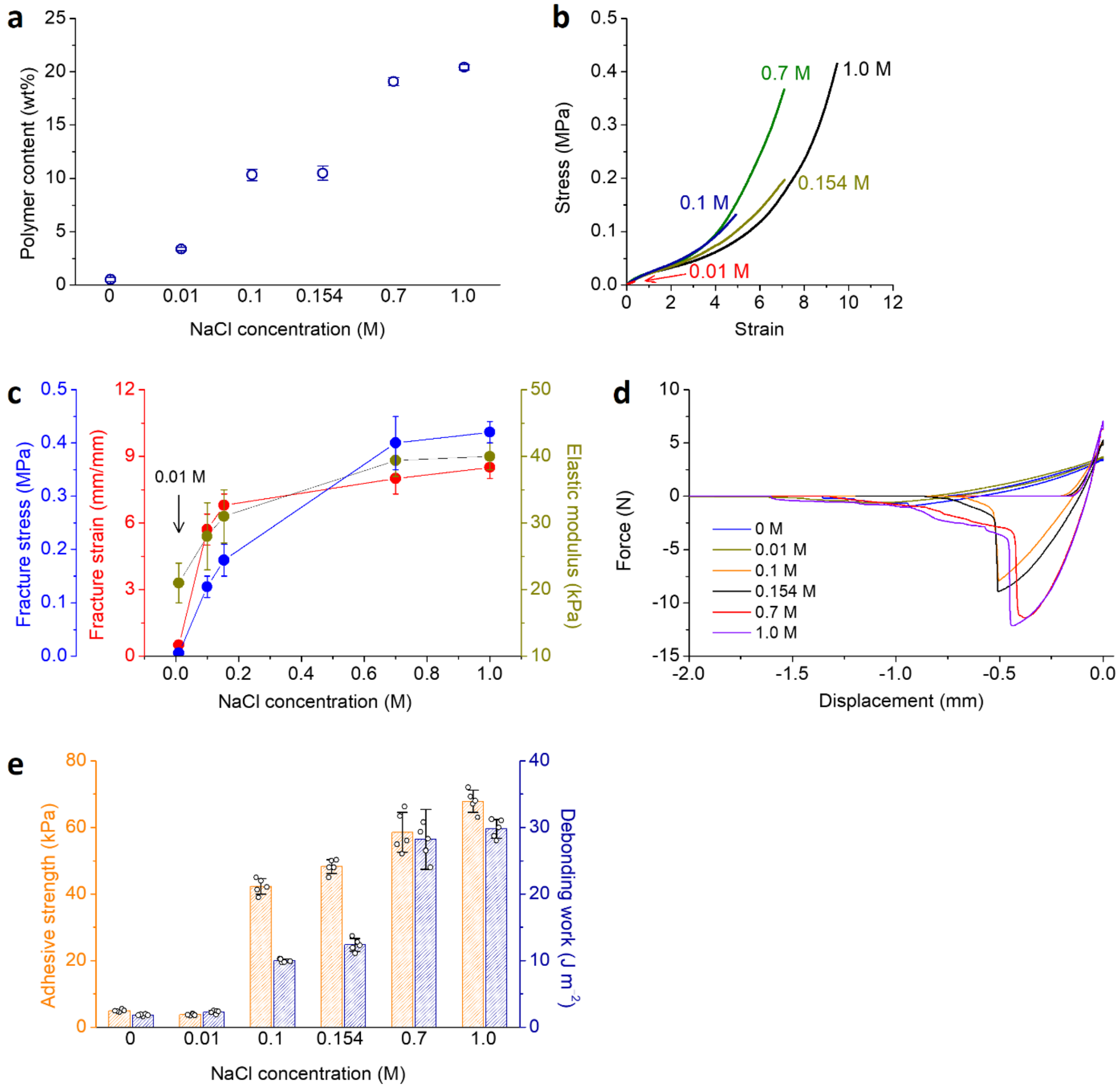


**Supplementary Figure 15.** Properties of P(ATAC-*adj*-PEA)-0.1 gels in a wide concentration range of NaCl solutions. **a,** Polymer contents. **b,** Stress–strain curves. **c,** The fracture stress, fracture strain, and elastic modulus of hydrogels obtained from stress–strain curves. **d,** Force–displacement curves of gel adhesion to the glass substrates. **e,** The adhesion values of the gel. The error bars indicate SD (N = 5). The P(ATAC-*adj*-PEA)-0.1 gel in pure water is too brittle for the tensile measurement; therefore, no mechanical data are shown in **b** and **c**. The tack tests were performed at a normal pressure equivalent to the elastic modulus $E$ of the gels.

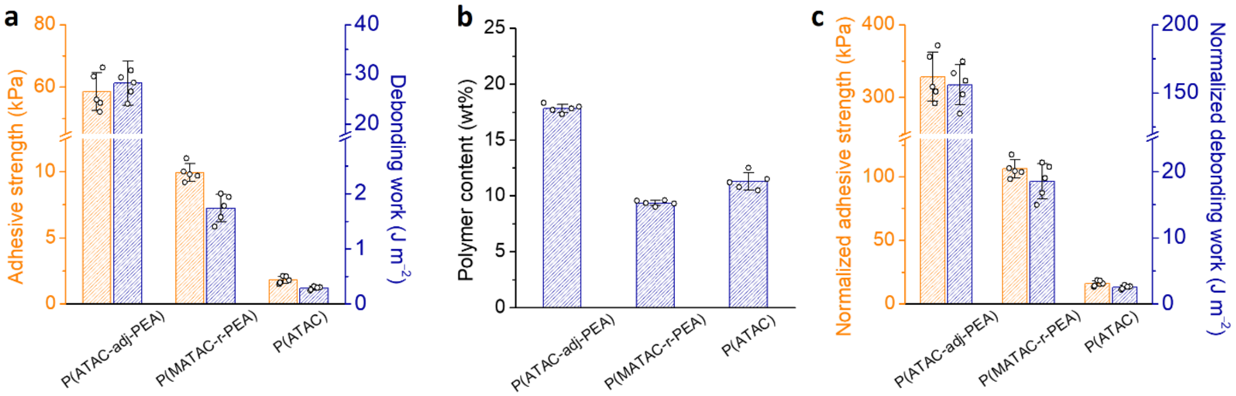


**Supplementary Figure 16.** Comparison of adhesion between P(ATAC-*adj*-PEA)-0.1 hydrogel and other control hydrogels to the negatively charged glass surfaces in a 0.7 M NaCl solution. **a,** The adhesion values of the hydrogels. **b,** Polymer contents of hydrogels. **c,** Normalized adhesion values of hydrogels. The error bars indicate SD (N = 5). All the tests were performed under 0.7 M NaCl solutions. Only the poly(cation-π) hydrogels that cationic and aromatic residues in adjacent position showed strong adhesion. The adhesion of other hydrogels were weak even taking consider their polymer contents. The normalized data was calculated by adhesive data divide by polymer content.

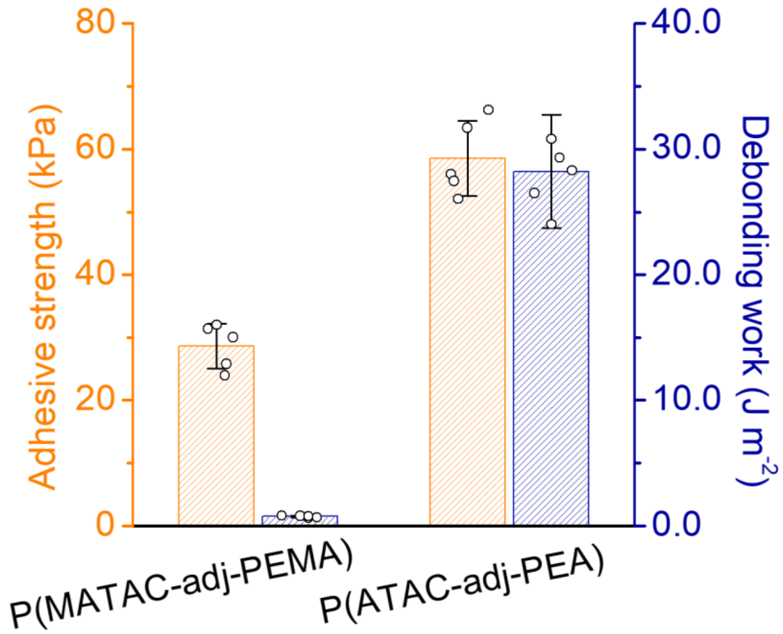


**Supplementary Figure 17.** Comparison of adhesion between the P(MATAC-*adj*-PEMA)-0.1 and P(ATAC-*adj*-PEA)-0.1 hydrogels to the negatively charged glass surfaces in a 0.7 M NaCl solution. The error bars indicate SD (N = 5).

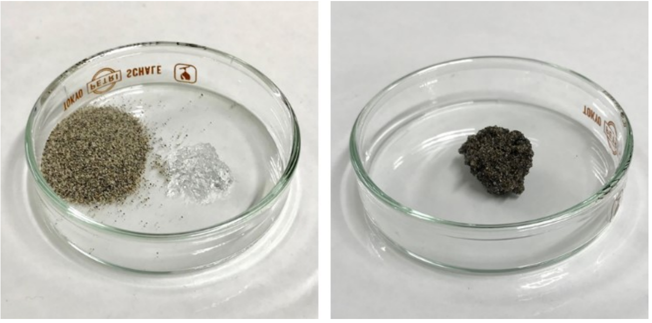

**Supplementary Figure 18.** Photographs of sea sand and highly viscous P(MATAC-*adj*-PEMA)/DMSO solution (left) and P(MATAC-*adj*-PEMA) glued sand (right). The gluing of sand was achieved by mixing the sand in the P(MATAC-*adj*-PEMA)/DMSO solution, and then washing it in seawater.

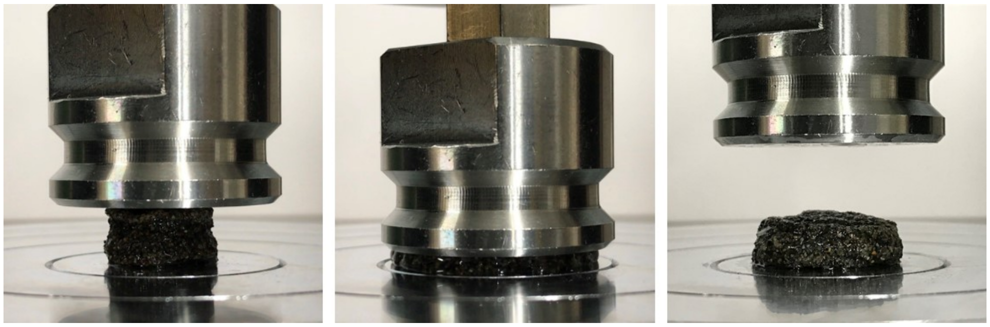

**Supplementary Figure 19.** Photographs of the P(MATAC-*adj*-PEMA) glued sand sustaining high compression and behaving like a tough elastic composite.

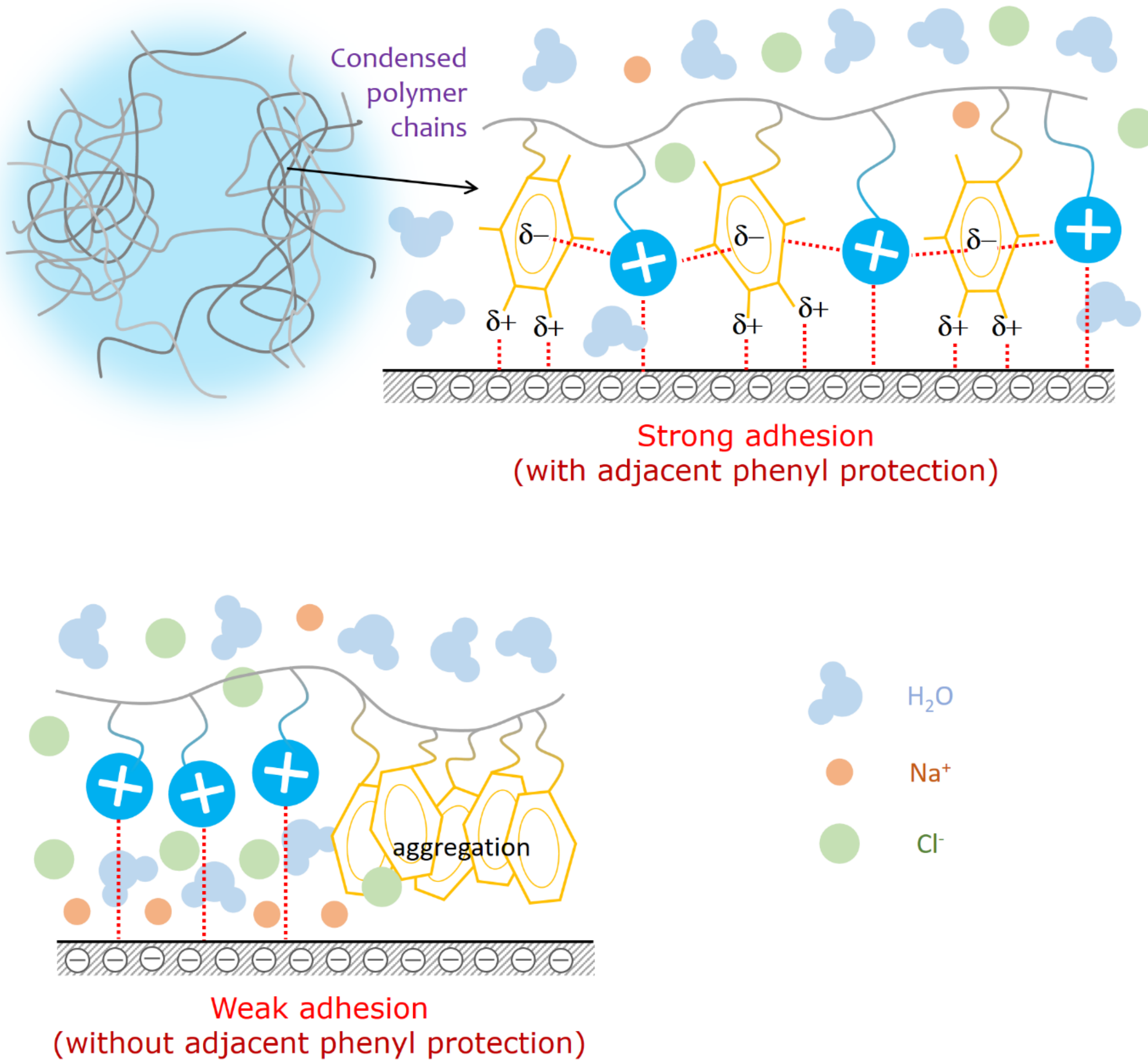


**Supplementary Figure 20.** Schematic illustration to show the proposed adhesion mechanism: at the molecular level, the cation–π interactions enhance the electrostatic interactions at the interface. At the microscale level, the coacervated structures trigger multiple electrostatic interactions, resulting in enhanced interfacial interaction. On the contrary, without adjacent phenyl protection, the electrostatic interactions between the cationic residues and the negatively charged surface will be weak.

**Supplementary Tables**

**Supplementary Table 1.** Formulation of samples synthesized in this study.

| Sample name | $C_m$ (M) | $C_x$ (mol%) | Polymer content (wt%)* |
|---|---|---|---|
| poly(cation-*adj*-π) hydrogels | | | |
| P(ATAC-*adj*-PEA) | 2.4 | 0 | 8.1±0.9 |
| P(ATAC-*adj*-PEA)-0.05 | 2.4 | 0.05 | 17.7±0.3 |
| P(ATAC-*adj*-PEA)-0.1 | 2.4 | 0.1 | 18.5±0.5 |
| P(ATAC-*adj*-PEA)-0.15 | 2.4 | 0.15 | 19.1±0.5 |
| P(ATAC-*adj*-BZA)-0.1 | 2.7 | 0.1 | 16.7±0.9 |
| P(ATAC-*adj*-PSEA)-0.1 | 2.4 | 0.1 | 20.5±1.2 |
| P(ATAC-*adj*-PDEA)-0.1 | 2.4 | 0.1 | 20.1±0.5 |
| P(APTC-*adj*-PEA)-0.1 | 2.2 | 0.1 | 14.6±0.6 |
| P(MATAC-*adj*-PEMA) | 2.2 | 0 | 27.4±0.2 |
| P(MATAC-*adj*-PEMA)-0.1 | 2.2 | 0.1 | 28.6±0.4 |
| poly(cation-*r*-π) hydrogels | | | |
| P(MATAC-*r*-PEA)-0.1 | 2.2 | 0.1 | 9.3±0.3 |
| P(ATAC-*r*-PEMA)-0.1 | 2.1 | 0.1 | 19.1±0.3 |
| control sample: poly(cation) systems | | | |
| P(ATAC)-0.1 | 2.4 | 0.1 | 11.5±0.9 |
| control sample: poly(cation-*co*-aliphatic) systems | | | |
| P(ATAC-*co*-MEA)-0.1 | 2.4 | 0.1 | 9.4±0.4 |

$C_m$: total in-feed monomer concentration in DMSO, $C_x$: density of chemical crosslinker MBAA relative to $C_m$. The monomers molar ratio is 1:1 in all copolymers. *In 0.7 M NaCl solution.

**Supplementary Table 2.** Formulation of hydrogels used as soft counter surfaces for adhesion tests of poly(cation-*adj*-π) hydrogels.

| Hydrogels | Monomer (M) | MBAA (mM) | 2-oxoglutaric acid (mM) |
|---|---|---|---|
| P(NaSS) *Negatively charged* | 1.4 | 140 | 3.5 |
| P(ATAC) *Positively charged* | 2.5 | 25 | 6.25 |
| P(AAm) *Neutral* | 3.2 | 3.2 | 8 |

## Supplementary References

1. Kim, S. Yoo, H. Y. Huang, J. Lee, Y. Park, S. Park, Y. Jin, S. Jung, Y. M. Zeng, H. Hwang, D. S. & Jho, Y. Salt Triggers the Simple Coacervation of an Underwater Adhesive When Cations Meet Aromatic π Electrons in Seawater. *ACS Nano* **11**, 6764-6772 (2017).

2. Poissant, R. R. Huang, Y. & Secco, R. A. A study of the sorbate–sorbent interactions in xylenes/zeolite Y systems by FT-Raman spectroscopy. *Microporous Mesoporous Mater.* **74,** 231-238 (2004).
