# Supplementary material for: Adjacent cationic–aromatic sequences yield strong electrostatic adhesion of hydrogels in seawater
Source: Nat Commun. 2019 Nov 12;10:5127. doi: 10.1038/s41467-019-13171-9 (PMC6851134; doi:10.1038/s41467-019-13171-9)
Supplement: Supplementary file 2 — Description of Additional Supplementary Files [file 41467_2019_13171_MOESM2_ESM.docx]

Description of Additional Supplementary Files

**Supplementary Movie 1**

A movie showing that the soft physical P(ATAC-adj-PEA) gel with a 15-mm diameter and 1.6-mm thickness can adhered to the negatively charged glass substrate strongly and underwent a large deformation during the retracting process before joint failure in 0.7 M saline water.

**Supplementary Movie 2**

A movie showing that the P(ATAC-adj-PEA)-0.1 gel with a 10-mm diameter and 1.24-mm thickness can rapidly adhere to a 0.49-kg glass block submerged in seawater, and the block can be lifted off of the seawater to air without failure of the adhesion
